# miR-200c Targets a NF-κB Up-Regulated TrkB/NTF3 Autocrine Signaling Loop to Enhance Anoikis Sensitivity in Triple Negative Breast Cancer

**DOI:** 10.1371/journal.pone.0049987

**Published:** 2012-11-21

**Authors:** Erin N. Howe, Dawn R. Cochrane, Diana M. Cittelly, Jennifer K. Richer

**Affiliations:** 1 Program in Cancer Biology, University of Colorado, Anschutz Medical Campus, Aurora, Colorado, United States of America; 2 Department of Pathology, University of Colorado, Anschutz Medical Campus, Aurora, Colorado, United States of America; Sun Yat-sen University Medical School, China

## Abstract

Anoikis is apoptosis initiated upon cell detachment from the native extracellular matrix. Since survival upon detachment from basement membrane is required for metastasis, the ability to resist anoikis contributes to the metastatic potential of breast tumors. miR-200c, a potent repressor of epithelial to mesenchymal transition, is expressed in luminal breast cancers, but is lost in more aggressive basal-like, or triple negative breast cancers (TNBC). We previously demonstrated that miR-200c restores anoikis sensitivity to TNBC cells by directly targeting the neurotrophic receptor tyrosine kinase, TrkB. In this study, we identify a TrkB ligand, neurotrophin 3 (NTF3), as capable of activating TrkB to induce anoikis resistance, and show that NTF3 is also a direct target of miR-200c. We present the first evidence that anoikis resistant TNBC cells up-regulate both TrkB and NTF3 when suspended, and show that this up-regulation is necessary for survival in suspension. We further demonstrate that NF-κB activity increases 6 fold in suspended TNBC cells, and identify RelA and NF-κB1 as the transcription factors responsible for suspension-induced up-regulation of TrkB and NTF3. Consequently, inhibition of NF-κB activity represses anoikis resistance. Taken together, our findings define a critical mechanism for transcriptional and post-transcriptional control of suspension-induced up-regulation of TrkB and NTF3 in anoikis resistant breast cancer cells.

## Introduction

Breast cancer is the most commonly diagnosed malignancy among American women, and an estimated 226,870 women will be diagnosed in 2012 [1]. Although the mortality rate for breast cancer has improved, it remains the second most deadly cancer for American women, with mortality largely attributed to metastatic disease [2]. Metastasis is a complicated process, during which cells must undergo dramatic phenotypic changes to migrate away from the primary tumor, survive in the vasculature or lymphatics, and finally colonize metastatic sites. Oncogenic epithelial to mesenchymal transition (EMT) is thought to play an important role in the ability of cells to acquire traits necessary to metastasize [3]. The miR-200 family of miRNAs has emerged as a potent regulator of EMT. miRNAs are small (18–25 nucleotide) non-coding RNAs that regulate gene expression post-transcriptionally by binding to the 3′ untranslated region (UTR) of the target mRNA [4], and inhibiting translation or targeting the mRNA for degradation [5]. The miR-200 family is comprised of two polycistronic clusters – miR-200c and miR-141 on chromosome 12 and miR-200b, miR-200a and miR-429 on chromosome 1. The miR-200 family is highly expressed in breast epithelial cells and luminal-like carcinomas, and lost in the more aggressive basal-like, or triple negative carcinomas [6], [7]. These miRNAs serve to maintain an epithelial phenotype and protect against EMT through repression of multiple targets, including the EMT-inducing transcription factors – ZEB1 and ZEB2 [8]–[10], genes involved in migration – FN1, MSN [11], and WAVE3 [12], and epigenetic regulators – SIRT1 [13], and Suz12 [14].

In addition to enhanced migratory and invasive capacity necessary for metastasis, cells must also resist anoikis while in transit to metastatic sites. Anoikis is apoptosis initiated by loss of attachment to the native extracellular matrix (ECM) [15], [16], and has been suggested as a physiological barrier to metastasis [16]–[19]. Anoikis resistance correlates tightly with an EMT phenotype [20]–[24]. We previously demonstrated that restoration of miR-200c to aggressive triple negative breast (TNBC) and Type 2 endometrial cancer cell lines significantly enhances anoikis sensitivity [11]. Furthermore, we identified the neurotrophic tyrosine kinase, type 2 (NTRK2 or TrkB) as a direct target of miR-200c, and demonstrated that addition of untargetable TrkB reversed the ability of miR-200c to sensitize TNBC cells to anoikis [11].

TrkB plays a crucial role in the formation and function of the nervous system [25], [26], including the promotion of neuronal survival [27]. TrkB was identified as a potent anoikis suppressor in a genome-wide screen for genes capable of conferring anoikis resistance to rat intestinal epithelial cells [28]. Indeed, TrkB and BDNF induce anoikis resistance in a variety of carcinoma models including breast [29], ovarian [30], [31], and head and neck [32]. However, neurotrophin 3 (NTF3) also activates TrkB [33], and is a predicted target of miR-200c. In this study, we present the novel finding that TNBC cells in suspension up-regulate both TrkB and NTF3 to enable anoikis resistance. We find that NF-κB drives transcription of both TrkB and NTF3, and that miR-200c potently suppresses anoikis resistance by directly targeting both components of this aberrant autocrine signaling loop.

## Results

### TNBC Cells are More Anoikis Resistant than Luminal A Cells and miR-200c Sensitizes TNBC Cells to Anoikis

We utilize four breast cancer cell lines to dissect miR-200c-mediated control of anoikis resistance. MDA-231 and BT549 cell lines are representative TNBC cell lines. They are motile and invasive in culture, and able to metastasize from an orthotopic site. Genetically they represent the basal B subtype [34]. They do not express estrogen receptor alpha (ERα), progesterone receptors (PR) or Her2/Neu, nor do they express the epithelial adherens junction protein E-cadherin or the miR-200 family, instead expressing mesenchymal markers such as N-cadherin and vimentin. MCF7 and T47D cells represent the luminal A subtype, expressing epithelial markers such as ERα, E-cadherin, and the miR-200 family. They are weakly invasive in culture and tumorigenic when provided with estrogen, but do not typically metastasize from the orthotopic site.

**Figure 1:**
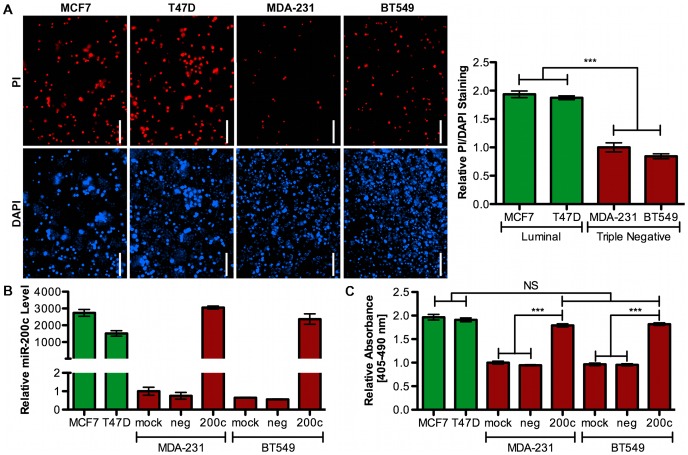
Triple negative breast cancer cells are more anoikis resistant than luminal cells and miR-200c sensitizes aggressive cells to anoikis. **A.** Cells were plated attached or suspended for 24 hrs prior to staining with DAPI and propidium iodide (PI). Representative images of suspended cells are shown, scale bar 50 µm. Quantitation of data in A, presented as a ratio of PI to DAPI, with each cell line normalized to the attached condition. Shown relative to MDA-231 cell line. *Columns*, mean of three biological replicates, *bars*, SEM. **B.** Cells treated with transfection reagent only (mock), scrambled negative control (neg) or miR-200c mimic (200c) and 48 hrs later harvested for qRT-PCR analysis of miR-200c levels. Data normalized to U6 levels and presented relative to MDA-231 mock transfection condition. *Columns,* mean of five biological replicates, *bars,* SEM. **C.** Cells as in B and 24 hrs later plated in suspension. After 24 hrs in suspension, a cell death ELISA was performed. Data normalized to attached condition and shown relative to MDA-231 mock transfection. *Columns*, mean of three biological replicates, *bars*, SEM.

We show that the less aggressive MCF7 and T47D cells are strongly sensitive to anoikis following 24 hrs in suspension, as demonstrated by strong staining with propidium iodide (PI) (Fig. 1A, Left). The MDA-231 and BT549 cells, however, show little apoptosis following culture in suspension (Fig. 1A, Left). When the proportion of PI to DAPI staining is quantitated, the luminal lines exhibit twice the amount of apoptosis observed in TNBC lines (Fig. 1A, Right). We next sought to determine if restoring miR-200c to the TNBC cells would sensitize them to anoikis to the extent observed in luminal lines. The levels of miR-200c attained following transfection of the mimic into the TNBC cells match the levels endogenously expressed in luminal lines (Fig. 1B). We find that restoration of miR-200c induces cell death in suspended TNBC cells (Fig. 1C). Taken together, this data indicates that miR-200c represses genes that TNBC cells require to resist anoikis, and expression of miR-200c in these cells sensitizes them to anoikis.

### TrkB Requires Ligand to Induce Anoikis Resistance

**Figure 2:**
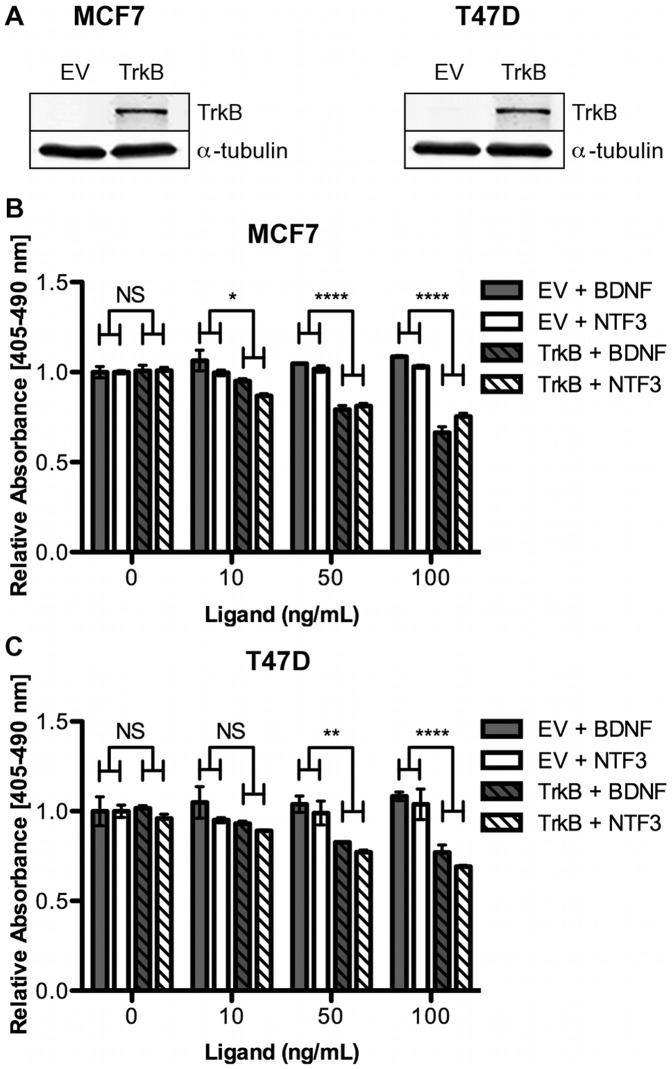
TrkB requires ligand to induce anoikis resistance. A. MCF7 and T47D cells were stably selected for expression of empty vector (EV) or TrkB. Immunoblot showing TrkB expression, α-tubulin used as loading control. MCF7, **B**, and T47D, **C**, cells were plated suspended in increasing concentrations of BDNF or NTF3. Cells were harvested 24 hrs later and apoptosis assayed by cell death ELISA, data normalized to attached condition and shown relative to EV conditions. *Columns*, mean of three biological replicates, *bars*, SEM.

TrkB is a cell surface receptor tyrosine kinase, and as such is activated by ligand binding. BDNF is the preferred ligand of TrkB in a neuronal setting [35]; however, it is not the only neurotrophic factor capable of activating TrkB. NTF3 also binds and activates TrkB [33], and NTF3 is a predicted target of miR-200c. To determine if BDNF or NTF3 activate TrkB signaling in a breast cancer model, we stably transfected empty vector (EV) or TrkB into MCF7 and T47D cells (Fig. 2A). The cells were then plated in suspension in medium containing increasing concentrations of BDNF and NTF3 (Fig. 2B, C). Addition of BDNF has no effect on EV expressing cells, but increases the anoikis resistance of TrkB expressing cells as expected, as indicated by decreased cell death. Likewise, treatment with NTF3 induces anoikis resistance of TrkB expressing cells to the same extent as BDNF. Of note is the fact that neither ligand affected survival of EV or TrkB expressing adherent cells (Fig. S1), indicating that activation of TrkB by BDNF or NTF3 affects anoikis specifically. Thus, in breast cancer cells, NTF3 is capable of activating TrkB to induce anoikis resistance to the same extent as BDNF, supporting our hypothesized role of NTF3 in TrkB-mediated anoikis resistance.

### NTF3 is a Direct Target of miR-200c

**Figure 3:**
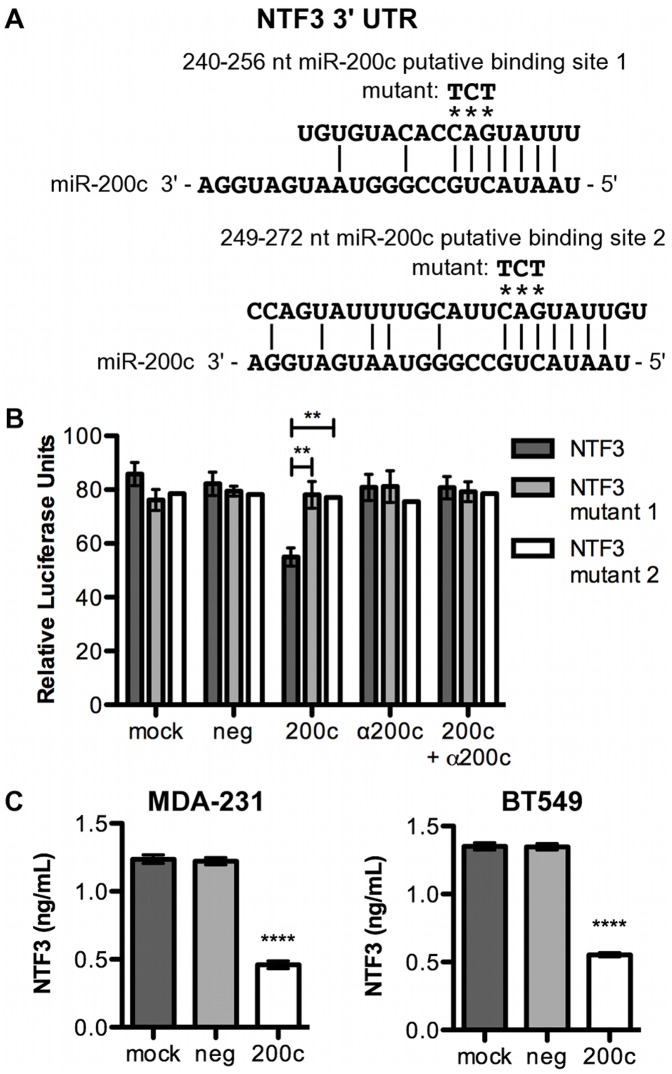
NTF3 is a direct target of miR-200c. A . Regions of the 3′ UTR where miR-200c is predicted to bind. **B.** Hec50 cells transfected with NTF3 luciferase constructs and 24 hrs later treated with transfection reagent only (mock), scrambled negative control (neg), miR-200c mimic (200c), miR-200c antagomiR alone (α200c) or in conjunction with miR-200c (α200c+200c) and luciferase assay performed. *Columns*, mean of five biological replicates, *bars*, SEM. **C**. Cells transfected with miRNA constructs and 48 hrs later medium collected for analysis by NTF3 ELISA. *Columns*, mean of three biological replicates, *bars*, SEM.

The 3′ UTR of NTF3 contains two putative miR-200c binding sites (Fig. 3A). We cloned the region containing these sites downstream of luciferase in a reporter plasmid. We observe a 35% decrease in luciferase activity following introduction of miR-200c, with no decrease in mock transfected or negative controls (Fig. 3B). When mutations are made in the putative miR-200c binding sites, luciferase activity returns to control levels, indicating that binding to either of these specific sites is required for down-regulation. When an antagomiR is used to inhibit miR-200c binding, luciferase activity is again restored. This indicates that miR-200c specifically is responsible for binding to the 3′ UTR. Together this data shows that miR-200c binds to two specific sites in the NTF3 3′ UTR to down-regulate reporter activity. Importantly, restoration of miR-200c to MDA-231 and BT549 cell lines leads to a significant decrease in the amount of secreted NTF3 (Fig. 3C). Thus, restoration of miR-200c to two TNBC cell lines significantly represses expression of NTF3 through direct targeting.

### miR-200c Suppresses Anoikis Resistance through Targeting of the TrkB/NTF3 Signaling Axis

**Figure 4:**
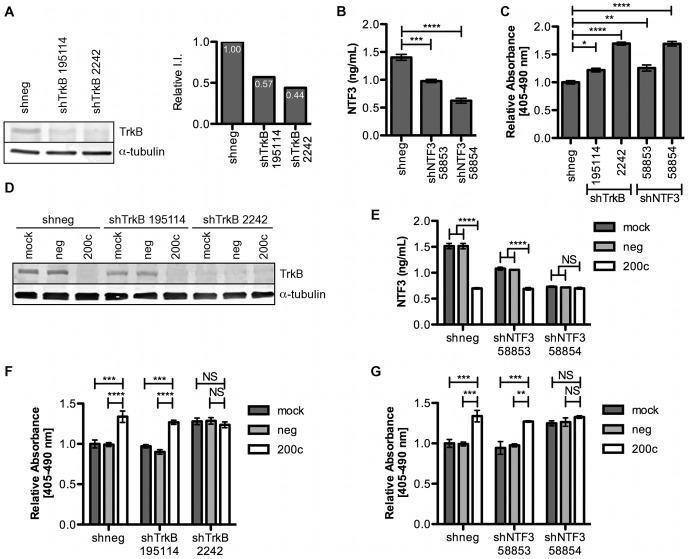
TrkB and NTF3 are required for anoikis resistance. BT549 cells stably selected for expression of shneg, shTrkB or shNTF3 constructs. **A.** Efficacy of TrkB knockdown. *Left*, immunoblot showing knockdown of TrkB, α-tubulin used as loading control, *right*, quantitation of immunoblot. **B.** Efficacy of NTF3 knockdown. NTF3 ELISA performed on medium. *Columns,* mean of three biological replicates, *bars*, SEM. **C.** Cell death ELISA performed on cells suspended for 24 hrs. *Columns,* mean of three biological replicates, *bars*, SEM. **D–G.** Cells treated with transfection reagent only (mock), scrambled negative control (neg) or miR-200c mimic (200c) and 24 hrs later plated in suspension. Cells were harvested 24 hrs later for analysis. **D.** Immunoblot for TrkB, α-tubulin used as loading control. **E.** NTF3 ELISA performed on medium. *Columns,* mean of three biological replicates, *bars*, SEM. shTrkB, **F**, and shNTF3, **G**, cells analyzed by cell death ELISA. *Columns,* mean of three biological replicates, *bars*, SEM.

Having shown that the combination of TrkB and NTF3 is sufficient to induce anoikis resistance in luminal breast cancer cells, we sought to determine if TrkB and NTF3 are necessary for TNBC cells to resist anoikis. To answer this question, we utilized shRNA constructs against TrkB and NTF3 to determine if knockdown of either component of the signaling loop would sensitize cells to anoikis. BT549 cells were stably selected for expression of shRNAs targeting TrkB or NTF3. shTrkB 2242 was most effective at knocking down TrkB expression with a 56% reduction (Fig. 4A), while shNTF3 58854 knocked down NTF3 most efficiently with a 60% reduction (Fig. 4B). To determine the effect of the shRNAs on anoikis resistance, cells were plated in suspension and harvested at 24 hrs. We show that expression of either shTrkB construct induces cell death, with shTrkB 2242 inducing a strong 80% increase in cell death, indicating a decrease in anoikis resistance (Fig. 4C). Similarly, both shNTF3 constructs decrease anoikis resistance, with shNTF3 58854 inducing the strongest decrease (Fig. 4C). Similar results were obtained in the MDA-231 cell line (Fig. S2). Overall, knockdown of either TrkB or NTF3 significantly decreased the ability of these cells to survive in suspension; thus, TrkB and NTF3 are necessary for BT549 and MDA-231 cells to resist anoikis.

To determine if suppression of TrkB and NTF3 is the mechanism by which miR-200c suppresses anoikis resistance, BT549 cells expressing shTrkB and shNTF3 constructs were transfected with miR-200c mimic and plated in suspension. We find that cells expressing the less effective shTrkB 195114 construct exhibit further repression of TrkB expression when transfected with miR-200c (Fig. 4D). Cells expressing shTrkB 2242 do not show further repression of TrkB when expressing miR-200c (Fig. 4D), indicating that miR-200c suppresses TrkB expression as effectively as shTrkB 2242. Similarly, cells expressing shNTF3 58853 secrete less NTF3 into the medium when transfected with miR-200c, while cells expressing shNTF3 58854 do not (Fig. 4E), again indicating that miR-200c suppresses NTF3 expression as effectively as shNTF3 58854. We next investigated the presence of additive effects between the shRNA constructs and miR-200c to determine if repression of TrkB and NTF3 signaling is the mechanism by which miR-200c suppresses anoikis resistance. We find that cells expressing shTrkB 2242 construct do not exhibit increased anoikis sensitivity following transfection with miR-200c (Fig. 4F), indicating that miR-200c suppresses anoikis resistance by specifically targeting a TrkB-mediated pathway. Supporting this hypothesis, shNTF3 58854 also does not exhibit increased anoikis sensitivity when transfected with miR-200c (Fig. 4G). Similar results were obtained in MDA-231 cells (Fig. S2). Since effective knockdown of either TrkB or NTF3 does not increase anoikis in suspended cells above that of miR-200c alone, we conclude that miR-200c suppresses anoikis resistance by targeting the TrkB/NTF3 signaling axis.

### TrkB and NTF3 are Up-regulated in Suspended Cells

**Figure 5:**
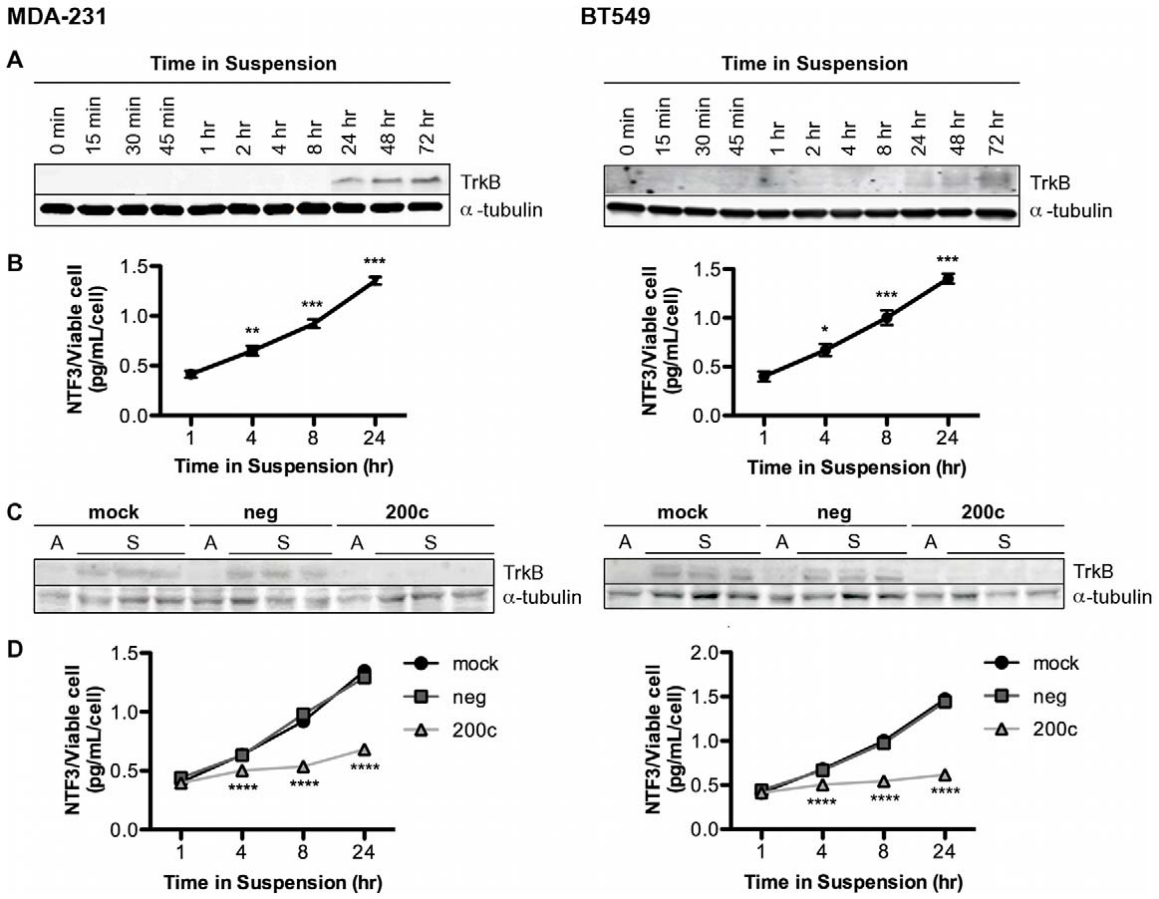
TrkB and NTF3 are up-regulated in suspended cells and miR-200c blocks this up-regulation. Cells were plated in suspension and harvested at the time points indicated. **A.** Immunoblot for TrkB expression, α-tubulin used as loading control. **B.** NTF3 ELISA performed on medium. *Points,* mean of three biological replicates, *bars*, SEM. Cells treated with transfection reagent only (mock), scrambled negative control (neg) or miR-200c mimic (200c) and 24 hrs later plated in suspension. **C.** Cells were harvested 24 hrs later and immunoblot performed for TrkB, α-tubulin used as loading control. **D.** NTF3 ELISA performed on medium at time points indicated. *Points,* mean of three biological replicates, *bars*, SEM.

We made the novel observation that both TrkB and NTF3 exhibit increased expression when TNBC cells survive in suspension. We examined TrkB protein in MDA-231 and BT549 cells following culture in suspension, and found dramatic up-regulation of TrkB beginning at 24 and persisting through 72 hrs in suspension in both cell lines (Fig. 5A). Furthermore, we found that the amount of NTF3 secreted into the medium, as determined by NTF3 ELISA, significantly increases in both cells lines as rapidly as 4 hrs in suspension (Fig. 5B). To determine the effect of miR-200c on suspension-induced up-regulation of TrkB and NTF3, cells were transfected prior to plating in suspension. We found that expression of miR-200c blocked suspension-induced up-regulation of both TrkB (Fig. 5C) and NTF3 (Fig. 5D). Together, this data indicates that anoikis resistant breast cancer cells dramatically up-regulate an autocrine signaling loop following loss of ECM attachment, and restoration of miR-200c blocks the ability to establish this loop. We next sought to determine if TrkB and NTF3 are up-regulated at the transcriptional and/or post-transcriptional level. TrkB and NTF3 mRNA levels increased as early as two hours (Fig. S3), indicating that the up-regulation of these genes is at the transcriptional level.

### NF-κB Transcriptional Activity Increases in Suspended TNBC Cells

**Figure 6:**
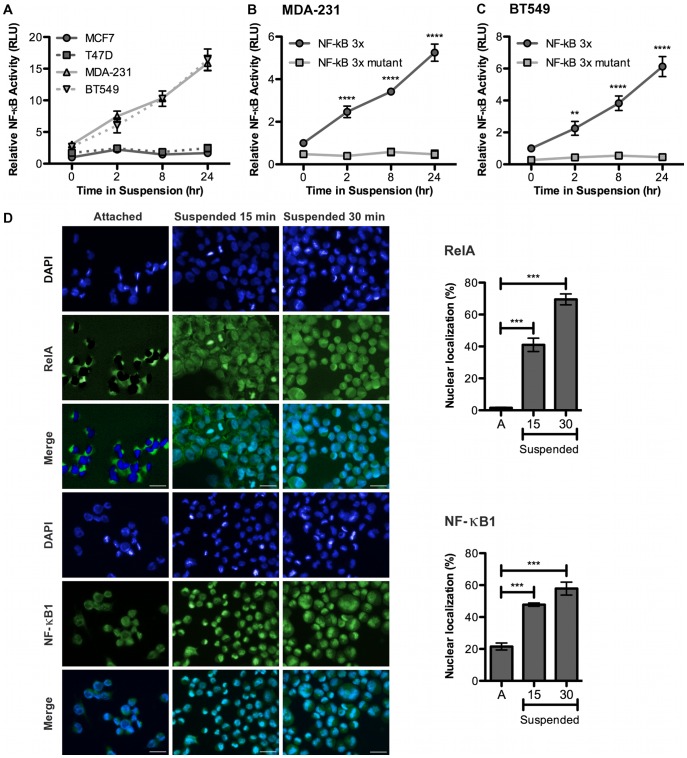
NF-κB transcriptional activity increases in suspended TNBC cells. A. Cells were transfected with 3x NF-κB transcriptional response element reporter and a *Renilla* control and 24 hrs later plated in suspension. Cells were harvested at time points indicated and dual luciferase assay performed. Data normalized to attached time point and presented relative to MCF7 attached condition. *Columns,* mean of three biological replicates, *bars*, SEM. MDA-231, **B**, and BT549, **C**, cells were transfected with 3x NF-κB or mutant reporter and assayed as in A. Data presented relative to NF-κB attached condition. *Columns,* mean of three biological replicates, *bars*, SEM. **D.** BT549 cells were grown on coverslips (attached), or in suspension and spun onto slides. Immunocytochemistry was performed for RelA or NF-κB1 (left), and the percentage of nuclear staining at each time point was quantitated (right). *Columns*, mean of three biological replicates, *bars*, SEM.

Examination of the promoter regions of TrkB and NTF3 revealed that both genes contain a number of predicted NF-κB binding sites. NF-κB transcription factors are held inactive in the cytoplasm until the inhibitory IκB complex is proteolytically degraded; thus, NF-κB signaling can be rapidly activated during conditions of cellular stress. Given that TrkB and NTF3 are up-regulated so quickly, NF-κB signaling was an attractive option to explore. To investigate NF-κB transcriptional activity we used a luciferase reporter containing 3 perfect NF-κB elements upstream of luciferase. We found that anoikis resistant MDA-231 and BT549 cells exhibit higher basal levels of NF-κB transcriptional activity than anoikis sensitive MCF7 and T47D cells, as indicated by increased luciferase activity (Fig. 6A). Importantly, NF-κB transcriptional activity increased dramatically only in the MDA-231 and BT549 cells during suspension (Fig. 6A). We show that the increased luciferase activity is specific to NF-κB transcriptional activity, since a mutant construct does not exhibit increased activity in the MDA-231 (Fig. 6B) or BT549 cells (Fig. 6C). To confirm these results, we investigated the cellular localization of two NF-κB transcription factors, RelA (p65) and NF-κB1 (p50), in BT549 cells. Both factors are largely cytoplasmic in attached cells, but translocate to the nucleus when cells are suspended, as indicated by co-localization of DAPI and RelA or NF-κB1 staining (Fig. 6D). Indeed, following 30 minutes in suspension, the percentage of nuclear RelA increases from 6% in attached cells to 70%, while the percentage of nuclear NF-κB1 increases from 21% to 58% (Fig. 6D, Right). Nuclear translocation of NF-κB factors is required for transcriptional activation; thus, this data suggests that these transcription factors are activated during anoikis resistance. Taken together, this data shows that the two NF-κB transcription factors predicted to target the TrkB and NTF3 promoters translocate to the nucleus following loss of ECM attachment, and there is enhanced NF-κB transcriptional activity under these conditions. This suggests that NF-κB transcriptional activity may be involved in suspension induced up-regulation of TrkB and NTF3.

### NF-κB Transcriptionally Up-regulates TrkB and NTF3 in Suspended Cells

**Figure 7:**
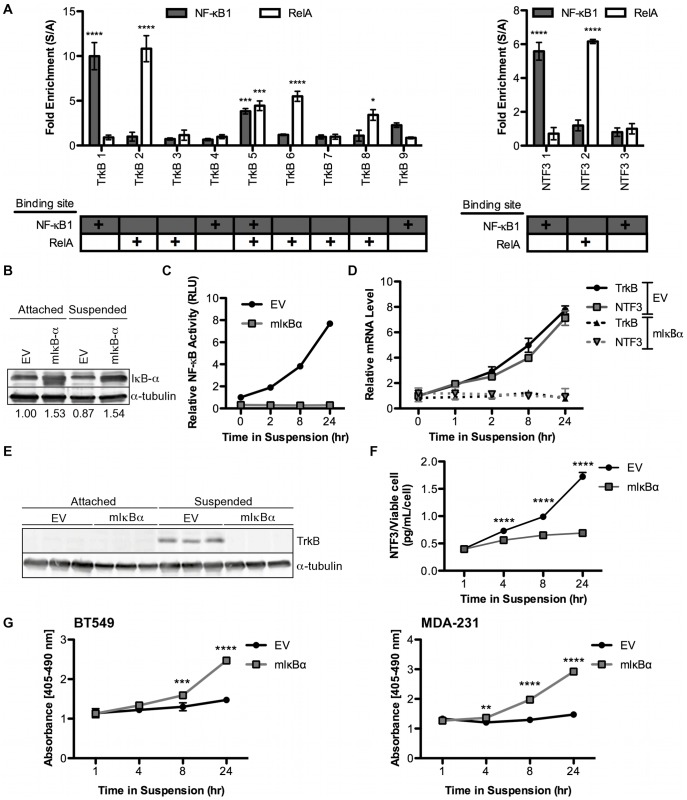
NF-κB transcriptionally up-regulates TrkB and NTF3 in suspended cells. A. BT549 cells were plated in suspension for 2 hrs and harvested for ChIP analysis. Following precipitation with antibodies against NF-κB1 and RelA, SYBR green qRT-PCR was performed for sites in the TrkB (left) and NTF3 (right) promoters. PLK1 used as a positive control for increased RelA binding in suspended cells. Data normalized to input controls and presented as a ratio of suspended over attached conditions. *Columns*, mean of three biological replicates, *bars*, SEM. **B–G** BT549 cells stably selected for empty vector (EV) or genetic NF-κB inhibition through mutant IκBα (mIκBα). **B.** Characterization of mIκBα cells, immunoblot of IκBα, α-tubulin used as loading control. Numbers represent amount of IκBα normalized to α-tubulin. **C.** Cells were transfected with 3×NF-κB transcriptional response element reporter and a *Renilla* control and 24 hrs later plated in suspension. Cells were harvested at time points indicated and dual luciferase assay performed. Data normalized to attached time point and presented relative to EV condition. *Points*, mean of three biological replicates, *bars*, standard error of the mean. **D.** Cells were plated in suspension and RNA was harvested at time points indicated. SYBR green qRT-PCR was performed for TrkB and NTF3. Data normalized to actin and presented relative to attached. *Points*, mean of three biological replicates, *bars*, SEM. **E.** Cells were plated in suspension for 24 hrs and harvested for immunoblot analysis of TrkB, α-tubulin used as loading control. **F.** NTF3 ELISA performed on medium at time points indicated. *Points,* mean of three biological replicates, *bars*, SEM. **G.** BT549 (left) and MDA-231 (right) cells were plated in suspension and harvested at the time points indicated for analysis by Cell Death ELISA. *Points,* mean of three biological replicates, *bars*, SEM.

To determine if RelA or NF-κB1 bind directly to regions in the promoters of TrkB and NTF3, and if binding increases when the cells are suspended, we performed chromatin immunoprecipitation (ChIP) on BT549 cells attached, or suspended for 2 hrs. Following IP using antibodies for RelA or NF-κB1, qRT-PCR was performed for 9 regions in the TrkB promoter and 3 in the NTF3 promoter that contain predicted binding sites for RelA or NF-κB1, as indicated by the chart (Fig. 7A, Bottom). Cycle thresholds were first verified to be above those seen in IgG controls (which were unchanged across conditions), and then normalized to the attached condition. The data is presented as a fold enrichment of PCR signal in suspended cells over attached cells, where a signal above 1 indicates that there is enhanced binding of the transcription factor in the region of being amplified. We show that sites 1 and 5 in the TrkB promoter exhibit increased NF-κB1 binding in suspended cells, while sites 2, 5, 6, and 8 exhibit increased RelA binding (Fig. 7A, Left). Site 1 in the NTF3 promoter exhibits increased NF-κB1 binding, while site 2 exhibits increased RelA binding (Fig. 7A, Right). This data demonstrates that in suspended cells, RelA and NF-κB1 translocate to the nucleus (Fig. 6D), and bind to specific regions in the promoters of TrkB and NTF3 (Fig. 7A).

To examine the effect of NF-κB inhibition on suspension-induced up-regulation of TrkB and NTF3, we utilized a mutant IκBα construct (mIκBα) that cannot be phosphorylated, and thus remains constitutively bound to NF-κB in the cytoplasm, preventing activation of NF-κB transcription. In empty vector expressing cells, IκBα levels decreased from 1.00 to 0.87 when cells are suspended, suggesting proteolysis of IκBα, such as would be expected during activation of NF-κB transcriptional activity. Cells expressing mutant IκBα exhibit no decrease in IκBα expression, as expected (Fig. 7B). Furthermore, expression of mutant IκBα prevents up-regulation of NF-κB transcriptional activity, as indicated by luciferase reporter activity (Fig. 7C). Next we sought to determine if inhibition of NF-κB signaling would inhibit up-regulation of TrkB and NTF3, and found that mutant IκBα completely repressed up-regulation of both TrkB and NTF3 at the mRNA level (Fig. 7D). Similarly, TrkB was not up-regulated in mutant IκBα expressing suspended cells (Fig. 7E), nor was soluble NTF3 (Fig. 7F). Taken together this data shows that two NF-κB transcription factors bind directly to the TrkB and NTF3 promoters, and that activation of NF-κB transcriptional activity is required for suspension induced up-regulation of TrkB and NTF3. Importantly, mutant IκBα sensitizes BT549 and MDA-231 cells to anoikis (Fig. 7G), demonstrating the full affect of the pathway, from the necessity of NF-κB transcriptional activation to anoikis sensitivity. Thus, NF-κB transcriptionally up-regulates TrkB and NTF3 through direct binding of RelA and NF-κB1 to the regions in the promoters, and this increased transcription is necessary and sufficient for triple negative breast cancer cells to resist anoikis.

## Discussion

Anoikis is not thought of as a classical component of EMT, but will perhaps soon be included since epithelial cells are sensitive to anoikis, while mesenchymal cells are not [36]–[38]. Fibroblasts require loss of matrix attachment coupled with growth factor depletion to induce anoikis [39], [40], while epithelial cells undergo anoikis even in the presence of serum. Additionally, resistance to anoikis is frequently observed in aggressive carcinoma cells, where it correlates with EMT [11], [20]–[24]. Loss of the miR-200 family of miRNAs also correlates with EMT, and we show that expression of miR-200c correlates with resistance to anoikis in breast cancer cell lines (Fig. 1). Further, restoration of miR-200c to basal-like breast cancer cells restores sensitivity to anoikis (Fig. 1) and [11].

Although many miRNAs have been found to influence EMT and MET [41]–[43], few have been shown to affect anoikis. Various molecular mechanisms are employed to achieve anoikis resistance, and the mechanisms differ between cell types [44], [45], complicating the identification of miRNA involvement. Hepatocellular carcinomas resist anoikis by expressing miR-221, which directly targets the pro-apoptotic protein Bmf [46]. Expression of miR-214 in melanoma cells promotes TFAP2C-mediated metastasis, mainly by promoting trans-endothelial migration, but also by suppressing anoikis resistance [47]. Finally, miR-451 suppresses anoikis resistance in non-small cell lung cancer [48], and miR-124 suppresses anoikis resistance in breast cancer [49], but molecular mechanisms (specific targets involved) remain to be identified. Our identification of TrkB [11] and NTF3 (Fig. 3) as direct targets responsible for the ability of miR-200c to restore anoikis sensitivity establishes it as a prominent miRNA-mediator of anoikis sensitivity. Further, we demonstrate that TrkB signaling is both necessary (Fig. 4), and sufficient (Fig. 2) for anoikis resistance in breast cancer. To our knowledge, this is also the first report of a miRNA targeting both components of an autocrine signaling loop to protect against the aberrant expression of the receptor-ligand pair in an inappropriate cell type.

**Figure 8:**
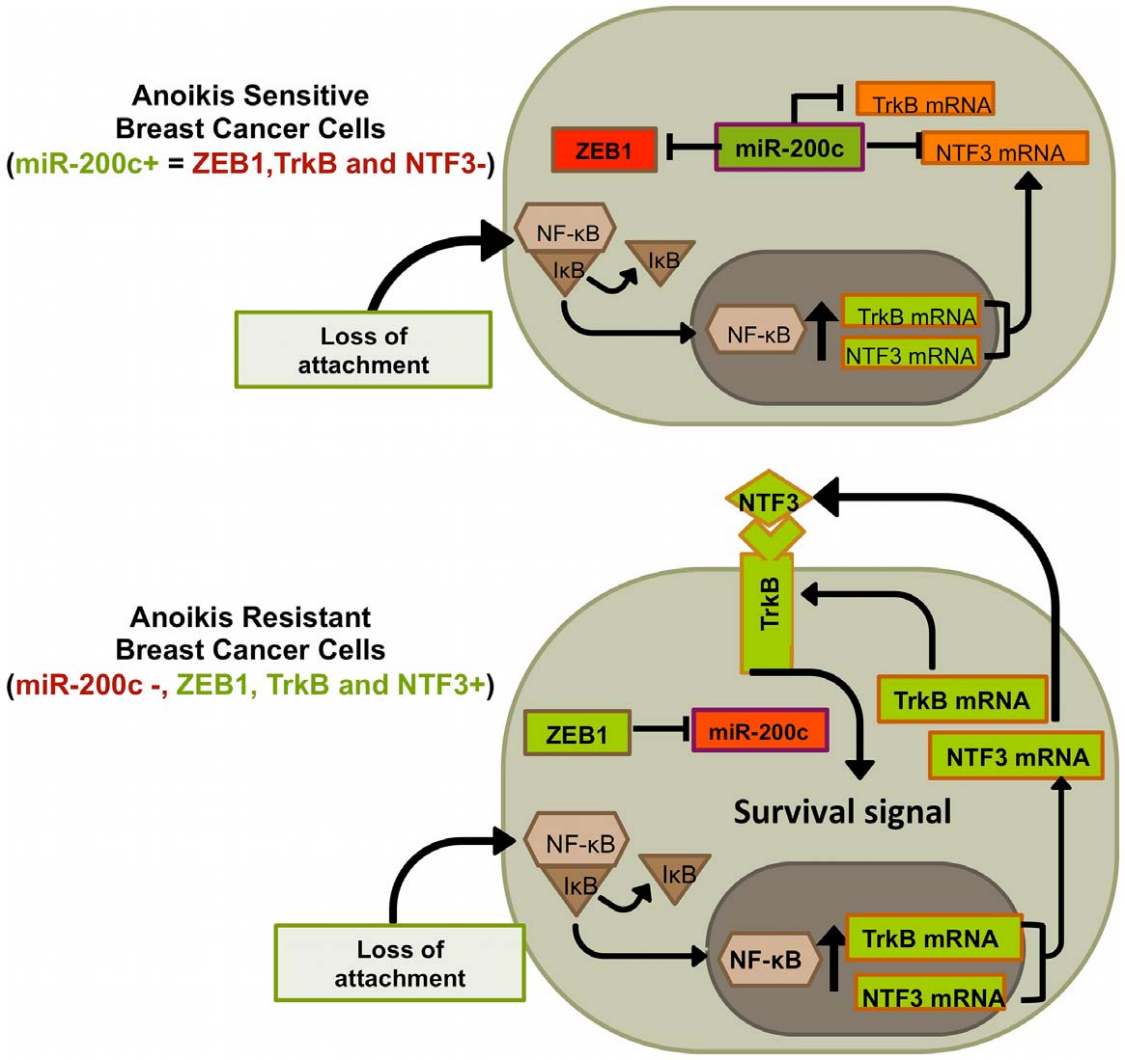
Model of select signaling pathways active in anoikis sensitive or resistant breast cancer cells. This model summarizes our findings regarding signaling pathways activated in breast cancer cells following loss of ECM attachment.

TrkB activated by exogenous BDNF confers resistance to anoikis [28]–[30], [50], and induces EMT through transcriptional activation of several EMT-inducing transcription factors [21], [22], [32]. We present the first evidence that, in breast cancer, endogenous NTF3 is secreted upon suspension to enable TrkB-mediated anoikis resistance. Transformation with TrkB acts through Snail and Slug to induce ZEB1 transcription [21], [22]. ZEB1 was recently found to be required for TrkB-induced EMT and anoikis resistance [22], and this likely due to the ability of ZEB1 to repress miR-200c [51], [52]. We previously demonstrated that miR-200c directly targets TrkB [11] and, as we show here, NTF3 (Fig. 3). Figure 8 is a diagram depicting these established interactions and our new findings. Collectively, the data suggest that transformation with TrkB may lead to repression of the miR-200 family, the loss of which helps maintain the transformed state. However, in the basal-like TNBC cells used in this manuscript, miR-200c is already extremely low to absent compared to luminal A ER+breast cancer cells (Fig. 1 and [6]). Even though miR-200c is absent, in the attached state these cells do not express detectable TrkB protein, but we demonstrate that upon detachment TrkB and NTF3 are up-regulated. This response is dependent on increased active NF-κB driving transcription of both TrkB and NTF3 combined with the fact that miR-200c is not there to repress translation of the transcripts into protein. Further supporting an important role for loss of miR-200c in anoikis resistant breast cancers, grainyhead-like-2 (GRHL2) was recently reported to oppose EMT and anoikis resistance through direct repression of ZEB1 [53] (which results in an increase in miR-200c). GRHL2 is lost in more mesenchymal-like breast cancers, such as TNBC and claudin-low, suggesting that signaling converging on ZEB1 and miR-200c are important not just for EMT, but also for anoikis.

The NF-κB family of transcription factors is composed of two classes, Class I, containing NF-κB1 (p50/p105) and NF-κB2 (p52/p100), and Class II, containing RelA (p65), RelB (p68) and c-Rel (p75) [54], [55]. NF-κB factors form hetero- or homodimers, which are held inactive in the cytoplasm. Thus, activation of NF-κB transcription requires only phosphorylation-induced degradation of the inhibitory IκB complex [56], [57]. NF-κB is best known for its regulation of innate and adaptive immunity [58], [59], where it was first discovered bound to the immunoglobulin promoter [60]. However, NF-κB also plays an important role in cancer, where it regulates proliferation and apoptosis [61], [62]. Our findings uniquely demonstrate that, in anoikis resistant breast cancer cells, NF-κB transcriptional activity increases to mediate direct up-regulation of TrkB, and its ligand, NTF3 setting up an aberrant autocrine signaling loop. Axctivated Trk family members signal through Akt to facilitate cell survival [32], [63]–[66]. Our finding that NF-κB up-regulates genes that facilitate anoikis resistance supports the appropriation of NF-κB signaling by cancer cells to avoid apoptosis. Specifically, our discovery of suspension-induced up-regulation of TrkB and NTF3 in breast cancer cells via NF-κB supports earlier work showing that polo-like kinase 1 (PLK1) is transcriptionally activated by RelA in suspended esophageal squamous cell carcinomas, leading to anoikis resistance [67]. Although few miRNAs have been implicated in anoikis, miR-125b has been found to be up-regulated in suspended mesenchymal stem cells, where it contributes to anoikis resistance through suppression of p53-mediated apoptosis [68]. This suggests that cells resist anoikis via different gene programs, depending on cell type of origin.

The mechanism by which NF-κB signaling is activated following loss of ECM attachment remains to be elucidated. However, integrin signaling is disrupted when the integrins are unligated to ECM components, yielding the possibility that integrin disruption activates NF-κB transcription. Integrins are obligate heterodimers, which link the ECM and the cytoskeleton. They are comprised of an α subunit, and a β subunit, and the specific α β composition dictates the ligand of the integrin [69]. Integrin-mediated activation of NF-κB signaling has been documented both in normal immune cell function, and in carcinoma models. However, the particular integrin mediating the activation, and the signaling program activated by NF-κB varies between systems. Neutrophils utilize α9β1 to avoid apoptosis through activated NF-κB signaling [70], while monocytes and monocyte-derived macrophages utilize αvβ3 to mediate a chronic inflammatory response [71]. In multiple myeloma, integrin β7 correlates with poor survival, activation of FAK, Src, and NF-κB signaling [72]. Various other integrin heterodimers have been found to activate NF-κB signaling in prostate cancer [73], melanoma [74], lung [75], and colorectal carcinoma models [76]. Interestingly, blockade of NF-κB signaling in gastric cancer prevents peritoneal dissemination of the disease through down-regulation of integrins α2, α3, and β1, which in turn prevents adhesion [77]. Future studies will determine if integrin signaling is responsible for the increased NF-κB induced upon detachment in breast cancer.

Finally, our identification of an autocrine signaling loop established by anoikis resistant breast cancer cells establishes a framework for exploration of combinatorial therapeutic strategies. Various strategies are employed by anoikis resistant cells, and many signal transduction pathways are concomitantly activated when cells should be committed to anoikis. Therefore, inhibition of multiple pathways should be the therapeutic aim, with a focus on avoiding activation of alternative pathways, and reducing toxicity. Because miR-200c targets genes involved not only in anoikis resistance [11], but in motility [8]–[12], proliferation [78], [79], chemoresistance [80]–[82], and stemness [14], [83], restoration of this miRNA along with an NF-κB inhibitor could serve as a potent combinatorial strategy.

## Materials and Methods

### Cell Culture and Treatments

MCF7 and T47D cells are available from the ATCC, and were grown in DMEM with 10% FBS, and 2 mM L-glutamine. MDA-231 cells are available from the ATCC, and were grown in MEM with 5% FBS, HEPES, NEAA, 2 mM L-glutamine, penicillin, streptomycin, and insulin. BT549 cells are available from the ATCC, and were grown in RPMI with 10% FBS and insulin. Hec50 cells were grown in DMEM with 10% FBS and 2 mM L-glutamine as described [84]. All cell line identities were verified by DNA profiling in the University of Colorado, DNA Sequencing and Analysis Core. Cells were treated with recombinant NTF3 (PeproTech, 450-03), and recombinant BDNF (PeproTech, 450-02) at the concentrations indicated.

### Transfection and Transduction

TrkB was subcloned from pBabe-TrkB (a gift from D. Peeper, Netherlands Cancer Institute) into pcDNA3.1 (Invitrogen). The 3×NF-κB and mutant 3×NF-κB were gifts from A. Baldwin (University of North Carolina). mIκBα was generously provided by R. Schweppe (University of Colorado, AMC). All shRNA constructs are part of the Sigma-Aldrich MISSION line, obtained from the University of Colorado, Functional Genomics Core – shneg (SHC002), shTrkB (TRCN0000195114, TRCN0000002242) and shNTF3 (TRCN0000058853, TRCN0000058854). miR-200c mimic or scrambled negative control (Ambion) were transfected at a concentration of 50 nM. Plasmids were transfected according to the manufacturer’s instructions. Lenti- and retroviral vectors were packaged in 293FT packaging cells (Invitrogen). All transfections were performed with Lipofectamine 2000 (Invitrogen) per the manufacturer’s instructions.

### Luciferase Assay

A section of the 3′ untranslated region (UTR) of NTF3 containing the putative binding sites for miR-200c was amplified by PCR from HeLa genomic DNA using the following primers, NTF3 F 5′ – CCACTAGTGCATGTAGCATA –3′, NTF3 R 5′ – CTCAAGCTTACAACAGTCAT –3′. Fragments were cloned into a firefly luciferase reporter vector (pMIR-REPORT, Ambion). Mutations were generated by PCR directed mutagenesis using the following primers, NTF3 mut1 F 5′ – TAAAATCTGTGTACACCATCTTTTTGC –3′, NTF3 mut1 R 5′ – TGACAAAGATGAATGCAAAATACTGGTG –3′, NTF3 mut2 F 5′ – TGCATTCATCTTTGTCAAGGCCATGACTGT –3′, NTF3 mut2 R 5′ – TGACAAAGATGAATGCAAAATACTGGTG –3′. Luciferase assay was performed on Hec50 cells using the Dual Luciferase Reporter assay system (Promega, E1960). NF-κB luciferase assays were performed on cell lines indicated, using the same DLR kit. All luciferase measurements are normalized to *Renilla* readings.

### Real-time Reverse Transcription-PCR

RNA was harvested from cells using Trizol (Invitrogen). SYBR Green real-time RT-PCR was performed using primers specific for each target, TrkB F 5′ – CCTGCTGGGTAGTGGCTGCG –3′, TrkB R 5′ – CATGGCATCCGTGTGGCCGT –3′, NTF3 F 5′ – CCTGCTGGGTAGTGGCTGCG –3′, NTF3 R 5′ – CATGGCATCCGTGTGGCCGT –3′, ACTIN F 5′ – CTGTCCACCTTCCAGGAGATG –3′, ACTIN R 5′ – CGCAACTAAGTGATAGTCCGC –3′. To avoid the possibility of amplification artifacts, PCR products for all SYBR Green primer pairs were verified to produce single products by agarose electrophoresis and high resolution melt curve. The relative mRNA levels were calculated using the comparative Ct method (ΔΔCt).

### Reagents

Primary antibodies used were TrkB (Cell Signaling, 4603S, 1∶1000), IκBα (Santa Cruz, sc-847, 1∶100), NF-κB1 (Abcam, ab7971, ChIP –2 µg/mL, ICC –1∶150), RelA (Abcam, ab7970, ChIP –2 µg/mL, ICC –1∶150), and α-tubulin (Sigma, clone B-5-1-2, 1∶30,000). Goat anti rabbit conjugated to Alexa Fluor 660 (Invitrogen, 1∶5000), and goat anti mouse conjugated to Alexa Fluor 660 (Invitrogen, 1∶5000) were used as appropriate, and signal was detected by Odyssey Infrared Imaging System (Licor Biosciences). For ICC, goat anti rabbit conjugated to Alexa Fluor 488 (Invitrogen, 1∶500) was used. NTF3 levels were detected by NTF3 ELISA (Promega, G7640).

### Anoikis Assays (Cell Viability and Cell Death ELISA)

Poly-hydroxyethyl methacrylate (poly-HEMA, Sigma-Aldrich) was reconstituted in 95% ethanol to12 mg/mL, and used to coat plates. For DAPI/PI staining, cells were stained with DAPI (Sigma-Aldrich, D8417, 20 µg/mL) and PI (Sigma-Aldrich, P4170, 1 µg/mL). For cell death, cells were harvested and assayed by cell death ELISA (Roche, 1 920 685).

### Digital Imaging

Images were collected using a Nikon ECLIPSE Ti system (Nikon). Quantitation was performed in ImageJ. Co-localization analysis was performed using the co-localization plug-in (http://rsbweb.nih.gov/ij/plugins/colocalization.html), which identifies pixels that exhibit fluorescence in both channels.

### Chromatin Immunoprecipitation

BT549 cells were harvested following 2 hrs in suspension, cross-linked, and chromatin extracted as described [85]. Samples were sonicated for 10 seconds 8 times on a Branson 250 Sonicator (Emerson). qRT-PCR was performed as described above using the following primers: NTF3 CHIP F1 5′ – gaaaagcagaacccgacaga –3′, NTF3 CHIP R1 5′ – cgcaagggtagggtagtcct –3′, NTF3 CHIP F2 5′ – cagggaggaaacgggatact –3′, NTF3 CHIP R2 5′ – agcagagttttgcccacttg –3′, NTF3 CHIP F3 5′ – acacacagcccctccctagt –3′, NTF3 CHIP R3 5′ – tagacccttccagctccaga –3′, TrkB CHIP F1 5′ – tgggtgattacgcacacact –3′, TrkB CHIP R1 5′ – ctgagctgcgcctctattct –3′, TrkB CHIP F2 5′ – agagccctcggaagtgtcag –3′, TrkB CHIP R2 5′ – tcctttaacctgacgggatg –3′, TrkB CHIP F3 5′ – gtgtgtgaactcccacatgc –3′, TrkB CHIP R3 5′ – caaaaacacacacacgctca –3′, TrkB CHIP F4 5′ – ggtgagcagcgcagatagt –3′, TrkB CHIP R4 5′ – taaaggggaatgcggagact –3′, TrkB CHIP F5 5′ – gaccagctcagcctctgata –3′, TrkB CHIP R5 5′ – catgccaccttatccaggac –3′, TrkB CHIP F6 5′ – aaagtgctgtgtgtatgttgtgtt –3′, TrkB CHIP R6 5′ – ggatgccatctcctaagcaa –3′, TrkB CHIP F7 5′ – gttgaaatgcactcgctcaa –3′, TrkB CHIP R7 5′ – caatgctaaagccagccttc –3′, TrkB CHIP F8 5′ – tgccaacgtagttgaccaag –3′, TrkB CHIP R8 5′ – atcctagcaccctggactca –3′, TrkB CHIP F9 5′ – tccaaagtctgtggcctttt –3′, TrkB CHIP R9 5′ – ccaccacacacacacaacaa –3′, PLK1 CHIP F 5′ – ccgtgtcaatcaggttttcc –3′, PLK1 CHIP R 5′ – cgtcctcgtccgctcaccat –3′.

### Statistical analysis

Statistical analysis was performed using GraphPad Prism 5. Student’s t-test, ANOVA with Tukey post-hoc test, and two-way ANOVA with Bonferroni multiple comparison test were used as appropriate. * p<0.05, ** p<0.01, *** p<0.001 **** p<0.0001, NS – not significant.

## Supporting Information

Figure S1 **TrkB signaling does not affect survival in attached cells.** MCF7 (top) and T47D (bottom) cells stably selected for expression of empty vector (EV) or TrkB were plated attached in increasing concentrations of BDNF or NTF3. Cells were harvested 24 hrs later and apoptosis assayed by Cell Death ELISA, data shown relative to EV conditions. *Columns*, mean of three biological replicates, *bars*, SEM.
(TIFF)

Figure S2 **TrkB and NTF3 are required for anoikis resistance.** MDA-231 cells stably selected for expression of shneg, shTrkB or shNTF3 constructs. **A.** Efficacy of TrkB knockdown. *Left*, immunoblot showing knockdown of TrkB, α-tubulin used as loading control, *right*, quantitation of immunoblot. **B.** Efficacy of NTF3 knockdown. NTF3 ELISA performed on medium. *Columns,* mean of three biological replicates, *bars*, SEM. **C.** Cell death ELISA performed on cells suspended for 24 hrs. *Columns,* mean of three biological replicates, *bars*, SEM. **D-G.** Cells treated with transfection reagent only (mock), scrambled negative control (neg) or miR-200c mimic (200c) and 24 hrs later plated in suspension. Cells were harvested 24 hrs later for analysis. **D.** Immunoblot for TrkB, α-tubulin used as loading control. **E.** NTF3 ELISA performed on medium. *Columns,* mean of three biological replicates, *bars*, SEM. shTrkB, **F**, and shNTF3, **G**, cells analyzed by cell death ELISA. *Columns,* mean of three biological replicates, *bars*, SEM.
(TIFF)

Figure S3 **TrkB and NTF3 up-regulation is transcriptional.** Cells were plated in suspension and RNA was harvested at time points indicated. SYBR green qRT-PCR was performed for TrkB and NTF3. Data normalized to actin and presented relative to attached time point. *Points,* mean of three biological replicates, *bars*, SEM.
(TIFF)
