# Supplementary figures and images for: miR-200c Targets a NF-κB Up-Regulated TrkB/NTF3 Autocrine Signaling Loop to Enhance Anoikis Sensitivity in Triple Negative Breast Cancer
Source: PLoS One. 2012 Nov 21;7(11):e49987. doi: 10.1371/journal.pone.0049987 (PMC3503774; doi:10.1371/journal.pone.0049987)

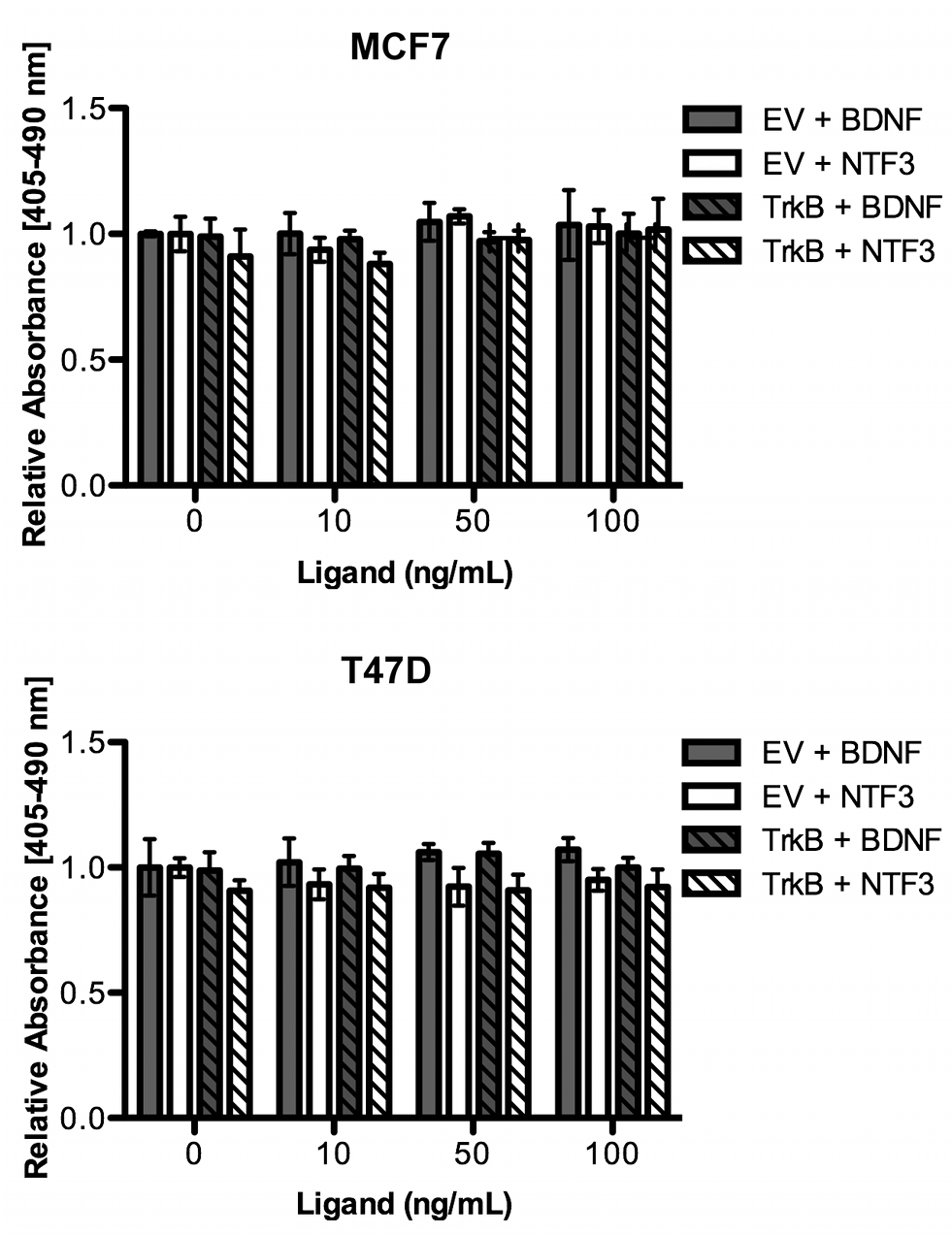

Supplement: Figure S1 — TrkB signaling does not affect survival in attached cells. MCF7 (top) and T47D (bottom) cells stably selected for expression of empty vector (EV) or TrkB were plated attached in increasing concentrations of BDNF or NTF3. Cells were harvested 24 hrs later and apoptosis assayed by Cell Death ELISA, data shown relative to EV conditions. Columns, mean of three biological replicates, bars, SEM. (TIFF) [file pone.0049987.s001.tiff]

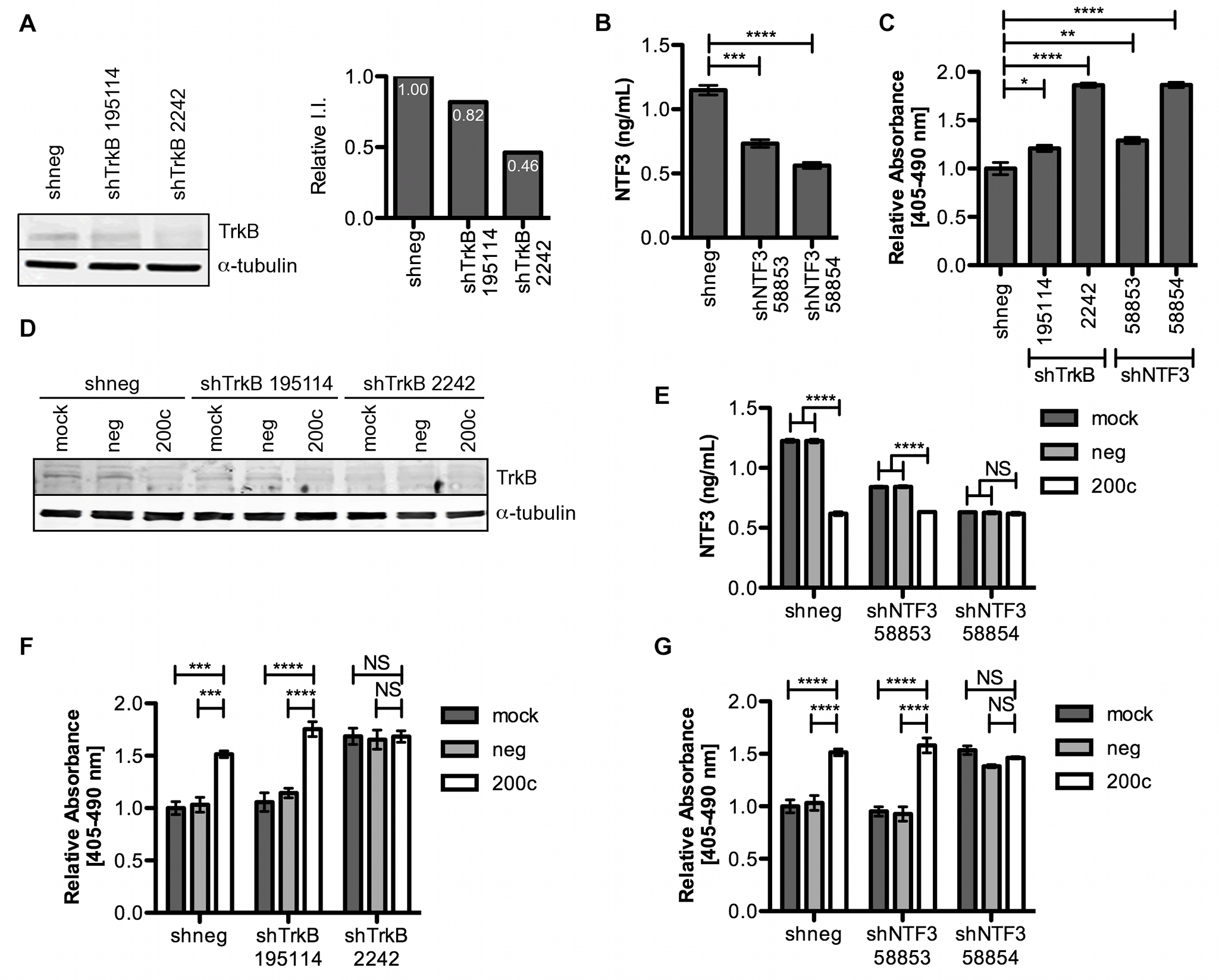

Supplement: Figure S2 — TrkB and NTF3 are required for anoikis resistance. MDA-231 cells stably selected for expression of shneg, shTrkB or shNTF3 constructs. A. Efficacy of TrkB knockdown. Left, immunoblot showing knockdown of TrkB, α-tubulin used as loading control, right, quantitation of immunoblot. B. Efficacy of NTF3 knockdown. NTF3 ELISA performed on medium. Columns, mean of three biological replicates, bars, SEM. C. Cell death ELISA performed on cells suspended for 24 hrs. Columns, mean of three biological replicates, bars, SEM. D-G. Cells treated with transfection reagent only (mock), scrambled negative control (neg) or miR-200c mimic (200c) and 24 hrs later plated in suspension. Cells were harvested 24 hrs later for analysis. D. Immunoblot for TrkB, α-tubulin used as loading control. E. NTF3 ELISA performed on medium. Columns, mean of three biological replicates, bars, SEM. shTrkB, F, and shNTF3, G, cells analyzed by cell death ELISA. Columns, mean of three biological replicates, bars, SEM. (TIFF) [file pone.0049987.s002.tiff]

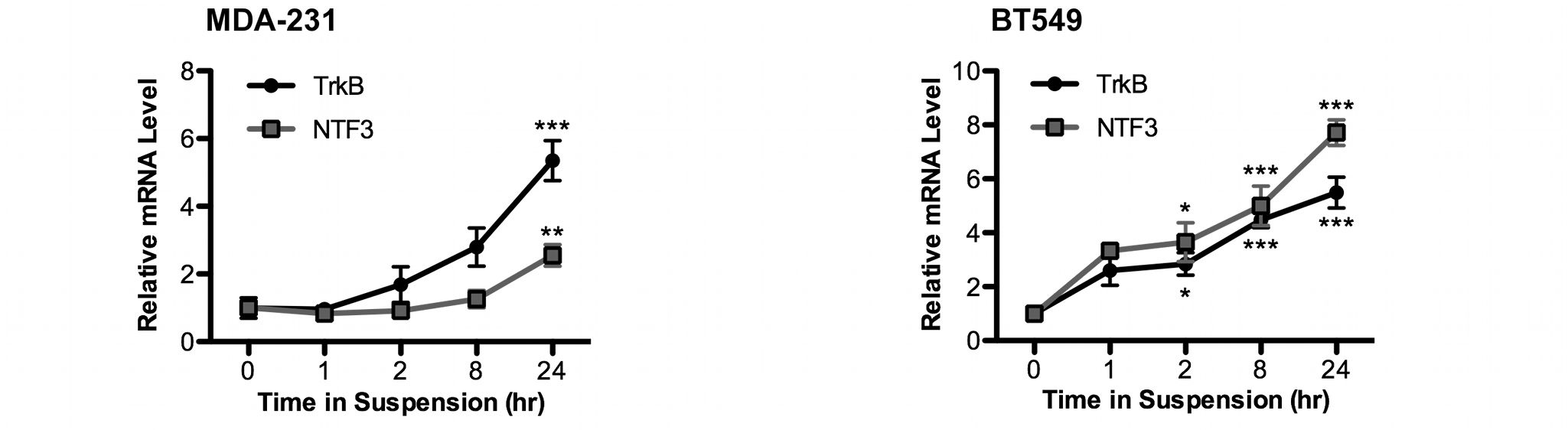

Supplement: Figure S3 — TrkB and NTF3 up-regulation is transcriptional. Cells were plated in suspension and RNA was harvested at time points indicated. SYBR green qRT-PCR was performed for TrkB and NTF3. Data normalized to actin and presented relative to attached time point. Points, mean of three biological replicates, bars, SEM. (TIFF) [file pone.0049987.s003.tiff]
